# Newly employed nurses’ transition into their new role in the ambulance service– a qualitative study

**DOI:** 10.1186/s12912-024-01745-y

**Published:** 2024-02-04

**Authors:** Klara Jepsen, Veronica Lindström, Jonas Wihlborg, Anna HÖrberg

**Affiliations:** 1 Department of Neurobiology, Care Sciences and Societ, Karolinska Institutet, Stockholm, Sweden; 2 Samariten ambulance AB, Stockholm, Sweden; 3 Department of Nursing, Umeå University, Umeå, Sweden; 4 Department of Ambulance Service, Region Västerbotten, Umeå, Sweden; 5 School of health and welfare, Dalarna University, Dalarna, Sweden

**Keywords:** Ambulance service, EMS, Introduction, Newly employed, Nurses, Transition

## Abstract

**Background:**

Nurses are essential to ensure safe and high-quality care worldwide. The World Health Organization (WHO) forecasts a shortfall of 5.9 million nurses by the year 2030, and in the ambulance service, the turnover rate ranges between 20% and 30%. With this study, we seek to increase knowledge by exploring the transition of newly employed experienced nurses into their roles in the ambulance service using the Meleis theory of transition. Through understanding transition, support for newly employed nurses can be developed, turnover rates can decrease, and in the long term, patient safety may increase.

**Design:**

The study employed a qualitative approach.

**Methods:**

Eighteen newly employed experienced nurses were individually interviewed four times during their first six months of employment. Deductive qualitative content analysis was used to analyse the data. The reporting of this research adheres to the COREQ checklist.

**Results:**

The results show that the transition process for newly employed nurses in the ambulance service encompassed all five aspects of Meleis’ transition theory: *Awareness, Engagement, Change and Difference, Time Span, and Critical Points*. The transition period varied among the participants, and it was also observed that not all nurses went through a transition in line with Meleis’ theory. Additionally, there were findings that nurses highlighted the impact of the ambulance service culture on their transition.

**Conclusions:**

The findings provide a more profound insight into how newly employed nurses with previous experience as nurses navigate their roles and transition into a new profession in a new context. An ambulance service where the organisation is aware of the newly employed nurses’ transition processes and what the transition entails can develop and promote a supportive and permissive culture within the ambulance service. For newly employed nurses who are adequately supported, health transitions are more likely to occur, which may increase retention and in the long term increase patient safety. The insights gained from the study can empower ambulance organisations to improve their introduction programmes and offer enhanced support for newly employed experienced nurses entering the ambulance service.

**Impact (Addressing:)**

- What problem did the study address?

The lack of knowledge and the transition process among the newly employed nurses with previous experiences as nurses during their introduction to the ambulance service.

- What were the main findings?

The experiences of the newly employed nurses transition in the ambulance service during their first year and their transition. All parts of Meleis theory were described by most of the nurses, implying that the theory can be used to explain and further understand new employees’ transition in the ambulance service. Additionally, the culture in the ambulance service affects the new employees, their introduction, and work satisfaction.

- Where and on whom will the research have an impact?

Understanding how newly employed nurses transition into their new role in the ambulance service can help educational institutions, ambulance organisations, and managers to improve introduction programs. Supporting newly employed nurses to healthy transition to a new profession can increase patient safety and increase retention.

**What does this paper contribute to the wider global clinical community?**

- Increased knowledge about how experienced nurses experience a change of workplace, introductory training, and developing their professional role in a new context.
- The knowledge that culture has an impact on the transition of newly employed nurses into a new working environment.
- Emergency Medical services constantly need to introduce new clinicians to the ambulance service and out on this study’s findings they can adjust the introduction programs to support the new employees’ transition.

## Introduction

Nurses play an essential role in the Emergency Medical services (EMS), ensuring the provision of safe and high-quality care [1]. At present, the WHO forecasts a shortfall of 5.9 million nurses by the year 2030 and emphasises the importance of retaining experienced nurses in the workforce [1]. Today, the turnover rate in the ambulance service, a part of the EMS, ranges between 20 and 30% [2, 3]. Being a newly employed nurse in a new context such as the ambulance service, with unpredictable care environments and a great variety of patient symptoms and illnesses, can evoke both excitement and nervousness [4]. Without sufficient support, nervousness and emotional distress represent a potential risk for turnover [2]. Consequently, gaining a better understanding of the transitional experiences of Registered Nurses (RN) and specialist nurses (SN) in the ambulance service is crucial for developing support and increasing the retention of new RNs as they enter their new professional roles. It is also known that new employees’ training, skills, and educational level are related to patient safety and patient outcomes in hospital as well as in the ambulance service, therefore it is important to reduce the risk of turnover [4, 5].

## Background

In health care services, the term ‘new employee’ is used for those who have been employed or newly trained within the previous 12 months [6–8]. Being newly employed can manifest in three different situations: the newly graduated nurse, the experienced nurse in a new context, and the experienced nurse in a new professional role [9, 10]. A nurse who is experienced but has changed their work setting may face similar feelings and challenges as a newly graduated nurse, such as concerns and uncertainty about learning new skills and routines [11]. An experienced nurse in a new professional role may also experience vulnerability and frustration in adapting to and feeling comfortable in the new working culture [12, 13].

Being new often involves a transition, defined as when a person undergoes significant changes and develops a new identity, attitude, or behaviour [14]. Transition can be described as a passage from one stage or condition to another [15]. Research shows that transition is a dynamic process and takes place over time [16–18]. An individual’s transition can be affected both positively and negatively by others, and different individuals need different types of support to succeed in their transition [10, 19, 20]. Furthermore, transition is a process that is greatly influenced by social interaction and the social context in which the individual is situated [21].

The ambulance service, a crucial component of the EMS, is primarily designed to provide care for people in the event of accidents or acute illnesses. Nonetheless, the ambulance service also encompasses a range of other responsibilities and care encounters that are less urgent, but more complex to handle [22]. In addition, the ambulance service is distinct from other healthcare settings due to the unpredictable working environment in which care is provided [23]. Patients may need to be cared for in their homes, at the scene of an accident, or inside a moving ambulance with limited access to hospital resources [4, 24]. Care in unsafe environments, linked with the need to make rapid decisions, particularly in unfamiliar situations, can result in increased levels of stress and a sense of vulnerability among the staff as well as increased risk for patient safety issues [25–27].

The first year of being new to the ambulance service has been proven to be a challenging period where new employees need extensive support [4]. A transition theory can be used to describe the journey of being new while becoming more secure in the new professional role. To the best of our knowledge, the transition of new employees in the ambulance service has not been previously explored. The transition theory presented by Meleis [15] describes five aspects of transition: awareness, engagement, change and difference, time span, and critical points. The theory has been used in the context of care, describing transitions for both patients and nurses [19]. By using Meleis’ transition theory [15] to explore and describe the process newly employed nurses go through while they are new to the ambulance service, the results of this study can provide a more profound insight into the transition process and how to support new employees in their professional development, ultimately improving the introduction programme.

## The study aim

The study aimed to explore the transition of newly employed experienced nurses during their introduction to the ambulance service, using Meleis’ transition theory.

## Methods

### Design

A qualitative design was used to explore the transition of newly employed experienced nurses during their introduction to the ambulance service, using Meleis’ transition theory [15]. The study conforms to the COREQ checklist for reporting qualitative research [28].

### Theoretical framework

To explore the transition of newly employed experienced nurses as they develop their new professional roles within the ambulance service, the theoretical framework of Meleis’ transitions theory was applied. According to Meleis, transitions are multidimensional and complex, and have several crucial properties [15]. These include *awareness, engagement, change and difference, time span, and critical points*. *Awareness* is related to knowledge and recognition of a transition experience. To be able to say that someone is in a transition, the person needs to have a certain awareness of the changes that are taking place. *Engagement* is defined by the level at which a person shows engagement in the various processes that comprise a transition. Examples of when a person shows engagement are actively preparing learning activities, using role models, and independently searching for information. There is no engagement if the person has no awareness of transition. In other words, the level of engagement is influenced by the level of awareness. All transitions involve *change and difference*, although not all change is related to transition. Dimensions of change include the nature, temporality, perceived importance or severity, and personal familial, and societal norms and expectations. Change may be related to critical or unbalanced events, disruptions in relationships and routines, or ideas, perceptions, and identities. Transitions are the result of change but also result in change. All transitions are characterised by flow and movement over a *time span*. The time span is defined as the first signs of anticipation, perception, or demonstration of change. It may, however, be difficult to put boundaries on the time span of certain transition experiences. *Critical points* are characterised by a sense of stabilisation in new routines, skills, lifestyles, and self-care activities. A period of uncertainty marked by fluctuation, continuous change, and disruption in reality [15].

### Study setting

The study was carried out in the capital of Sweden, Stockholm. There are approximately 2.2 million residents in the capital and its suburbs. It is the regional county council that is responsible for the ambulance service in Stockholm and three different organisations are contracted by the county council to operate the ambulance service. Ambulances in Sweden are staffed with a RN with at least a bachelor’s degree and more than one year of working experience but in some regions (30%), there is also a competence requirement that one of the professionals in the ambulance must be a SN [29]. To become an SN, there is an additional one-year study programme at an advanced level (second cycle) that includes a one-year master’s degree [30]. The RNs and SNs work with each other, or the SN works with an emergency medical technician (EMT) with basic life support competencies [31]. In Stockholm, the ambulances need to be staffed with at least one SN and most commonly the SNs additional education includes a specialisation in ambulance, intensive, or anaesthesia care [32]. Every year approximately 22 RNs and 14 SNs start the introductory training in the ambulance service in Stockholm. The region of Stockholm stipulates the content and length of the introductory training, and the different ambulance organisations complete the newly employed nurses’ training. At present, there is one introductory training period every year. The introductory training period is approximately eight weeks long and the content includes three weeks of theoretical education, one week of driving training, and four weeks of practical training with a senior colleague as a supervisor. There are no formal requirements for supervisors but ideally the supervisor should have worked for at least one year in ambulance service and the newly employed SN should be supervised by another SN with at least one year of experience in the ambulance service. The supervisors are selected based on the managers’ assessment.

### Participants

A convenience sample of 18 newly employed experienced nurses who were assigned to receive their introductory training in the ambulance service between the years 2020 and 2022 were asked to participate in the study, and all agreed. All participants were newly employed at one of the organisations that are contracted by the region to operate the ambulance service. The participants consisted of 11 RNs and seven SNs, as shown in Table 1. Henceforth, RNs and SN are referred to as nurses. Of the participants, 15 (83%) were female, which aligns with the trend of increasing female employment in the ambulance service [33].

**Table 1:** Description of Participants

Gender	Total *n* = 18
Female	15 (83%)
Male	3 (17%)
Age - years	
Range	25–51
Mean/median	34/ 32
Degree	
Registered nurse	11 (61%)
Specialist nurse	7 (39%)
Working experience in healthcare (years)	
1–5	4 (22%)
6–10	7 (39%)
11–15	3 (17%)
>16år	4 (22%)

### Data collection

Individual telephone interviews were used for data collection to facilitate participants’ participation in the study. Each participant was scheduled for interviews four times during their first six months within the organisation. The first interview was conducted immediately after the end of the newly employed nurses’ theoretical introduction, the second interview after approximately three weeks, the third after eight weeks, and the final interview was conducted six months after the first interview. Data were collected during the period 2020–2022. The interview guide used consisted of four questions (Table 2) and was developed by the first author and reviewed by the co-authors.

**Table 2:** Interview guide

Questions
1. How have your first weeks in the ambulance service been? (First-time interview) How has it been since the last time we spoke?
2. How do you see your professional role right now?
3. Can you tell me a little about how you see your ability to cope with your tasks?
4. Describe your role in the group (In the ambulance, at the station, and in the organisation)?

To enable participants to schedule the interview at their convenience a text message was sent by the interviewer a week before the planned interview. The interviews lasted 7–34 min (mean 18 min) and were digitally recorded. A total of 44 interviews were conducted; four interviews were missed because of challenges encountered in coordinating the telephone calls. The 44 interviews were conducted equally by the first and second authors. The interviews were audio-recorded and transcribed verbatim.

### Data analysis

**Fig. 1:**
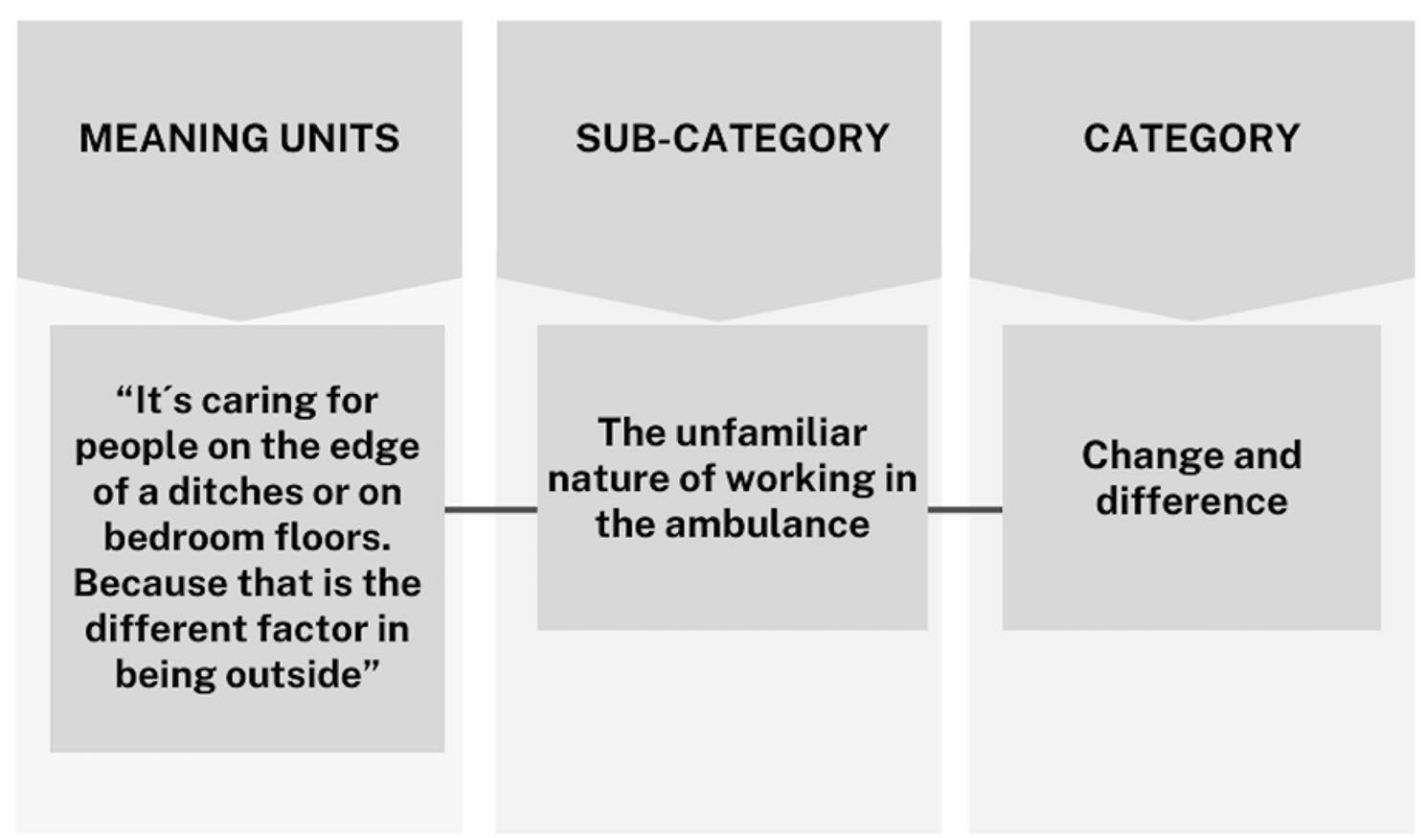
Example of analysis

A deductive qualitative content analysis was carried out in accordance with the three phases of *preparation, organisation, and presentation* presented by Elo and Kyngäs [33]. The transition theory described by Meleis was used as a framework and guided the analysis process. In the ***first phase***: *preparation*, all the recorded interviews were transcribed verbatim; thereafter, the transcribed data was read to get a sense of the whole. A categorisation matrix was developed based on the five elements of transition, *Awareness, Engagement, Change and difference, Time span, and Critical points* described by [15]. Meaning units regarding transition were identified and highlighted using different colour markings in the software program Atlas.fi. Meaning units that could not be sorted into the matrix but were related to the transition were sorted as “other” at this stage. ***Second phase***: Meaning units in each group were sorted and coded according to similarity and labelled with a descriptive word or sentence. The sorting process continued for as long as it was possible and reasonable. An example of the analysis process is shown in Fig. 1. The meaning units grouped as “other,” which did not align with the five elements of transition but included descriptions of the newly employed nurses’ transition, were also coded. They formed a sixth group dealing with addressing the ambulance culture. After the initial sorting, and grouping the analysis process followed an inductive approach [33], and 14 sub-categories were formed. ***In the third phase***: *Presentation*, the six categories, *Awareness, Engagement, Change and difference, Time span, Critical points, and Dealing with ambulance culture* were described, supported by the 14 sub-categories identified in the second phase of analysis. As transitions are multidimensional and complex, with several crucial properties, there are no distinct boundaries between the five aspects of the transition. For example, a person can display awareness of their transition through engagement. However, for clarity reasons, the five aspects of the newly employed nurses’ transition will be presented one by one in the results. During the whole analysis, the authors had continuous meetings and discussions, moving back and forth between the transcribed interviews, meaning units, sub-categories, and categories to preserve the newly employed nurses’ experiences.

## Results

**Fig. 2:**
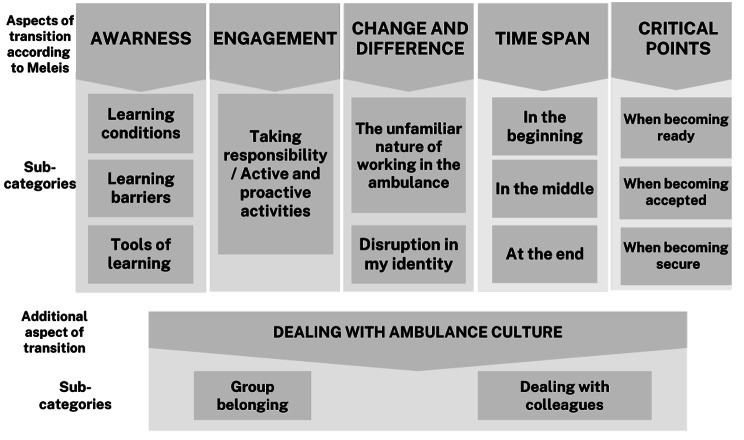
Illustration of results

Our results show that the transition process for newly employed nurses encompassed all five aspects of Meleis’ transition theory: *Awareness, Engagement, Change and Difference, Time Span, and Critical Points*. According to Meleis’ transition theory, a transition only occurs when the new individual shows awareness of change. Nurses who did not describe any awareness during the interview period (six months) were therefore considered not to have gone through a transition. The duration of the transition time varied and had no time limit or average time, as it depended on and differed among the nurses. For those nurses who showed a transition, all aspects of Meleis’ theory were identified. An additional finding during the analysis process was recognised, forming a sixth category—Dealing with Ambulance Culture, revealing that contextual culture seems to be an influential part of the transition for newly employed nurses. Each category will be further elaborated using the sub-categories presented in Fig. 2.

### Awareness

The category of awareness involves the newly employed nurses’ consciousness of their learning and development of a new professional role in a new context. This was displayed by the nurses’ descriptions of what adjustments or coping strategies they used to learn (*learning condition*), and what impact the context had on their learning (*learning barriers*). Awareness was also displayed when the new employees displayed a perception of change and talked about the significance of different learning strategies (*tools for learning).* The newly employed nurses displayed consciousness of learning by acknowledging what conditions for learning they identified in the ambulance service, such as telling themselves that they were in a learning process and that it was OK to be new and to ask for help.

I think it was the mental adjustment in myself, to be honest. I was prepared for the fact that now I’m new, so really. But just this feeling that oh, there’s so much I should be able to do, and I have no idea about this. I have never experienced this before. 12:5.

However, being new was also described as extremely challenging, and a *learning condition* to handle being new was to display humbleness before the change, by talking about this change of being new openly and acting in an unpretentious manner. Even though some aspects of working in the ambulance service were perceived as extremely difficult, the nurses also displayed awareness of the importance of having self-confidence in their ability to learn, and described this awareness of having self-confidence as a condition for learning. Furthermore, newly employed nurses were aware of and identified *barriers to learning*, such as self-doubt and fear, and utilised various tools for learning. For instance, peer support was emphasised as a facilitating *tool for learning*. Recognising prior knowledge and experiences was also perceived as a useful *tool for learning*. Finally, when displaying awareness, the nurses were cognizant of the different tools they used to move forward in their learning process, such as peer support as a facilitator for learning. Recognising prior knowledge and experiences, along with an increased contextual understanding facilitated their learning.

”I*t’s been good, I really like this peer learning thing. I am used to it and have seen it as a very positive way to learn 2:1”.*

### Engagement

This category refers to the active involvement and commitment made by the newly employed nurses in the process of transitioning from their previous profession to their new profession in the ambulance service. They displayed engagement by *taking responsibility* for their learning through *active actions* to handle different caring situations as well as *proactive actions* to prepare themselves for new caring situations.

*“I’m going to study the guidelines a little better and then it’s really just to get out and work. You learn to deal with this job and fill your experience bank as well as how to resolve different situations. 15:8”.*

Examples of active activities were using a new structured strategy to work, by actively choosing not to drive the ambulance so they could read medical guidelines on the way to the scene and/or working according to a structure such as A to E [, A = Airway, B = Breathing C = Circulation D = Disability E = Exposure]. The newly employed committed using structure, and role models such as supervisors, mentors, and colleagues if they were perceived as trustworthy in the caring situations, were described as especially useful when working with critically ill patients. The new nurses also had an active involvement in their learning by using proactive activities. By seeking feedback and follow-up on patients from the hospital and/or reading about the symptoms afterwards, aiming to increase their learning, the nurses displayed responsibility for their own learning in a new profession in a new context.

*“.so I ask them at the emergency room to contact me if there is something I have missed that is important. 24:4”.*

### Change and difference

The transition was shown in the narratives describing changes and differences acknowledged by newly employed nurses as they progressed from one state to another. This recognition involved the inherent alterations and distinctions within the transition processes. In the ambulance service, this was shown from two perspectives, i.e., the contextual change and the personal change. The *unfamiliar nature of the ambulance service* was emphasised by the newly employed nurses comparing the work to their prior workplaces. One difference was caring for only one patient at a time, which the nurses did not find stressful and they could give all their attention and time to the patient. In addition, the physical environment surrounding the patient was different from the patients in the hospital where the patient was always lying in a bed or on a chair in a well-lit examination room with all conceivable equipment within reach. In the new working environment, the patient might be in a ditch or on a bedroom floor, or in a moving vehicle with limited lighting.

It’s still new, but it’s not like this… Oh, we’re in a moving car and there’s no lighting. Not at all like hospital lighting. 12:7.

Furthermore, working in the ambulance service was described by the nurses as encompassing technical challenges, such as interacting through radio communication, using technical equipment, and driving the ambulance.

Another difference regarding the new and unfamiliar context was the level of available support and resources. The nurses said that in the ambulance service, they held the responsibility for both nursing and medical decisions, not a physician. Since they only worked with one colleague, they could not just push a button and help would be there within a few minutes. It could take a while for backup to arrive, which was described in contrasting terms, as giving both a sense of freedom, and fear, vulnerability, and exposure.

*“.many more own decisions as a nurse and I’m used to always having a doctor who makes the first assessments, and has the medical responsibility…so you don’t make these decisions in the hospital. It’s great fun to be able to make more decisions but it´s very new and so much responsibility. 2:24”.*

When talking about change and differences the nurses also talked about their own experience of being in a constant change as a *disruption in my identity*. During their introduction, the new employees experienced an information overload where the number of new things to learn was more than they had imagined. The nurses also experienced going from feeling like an expert at their previous workplace and always knowing what to do, to, as a newly employed person lacking the experience and knowledge needed to know what to do. This disruption of identity and the feeling of having to start learning from the beginning was described as emotional by the nurses.

You are on completely foreign ground; you have lost your footing a little bit from having been at the same workplace for 20 years. 7:9.

### Time span

The category of time span consists of the processes that occurred during the first six months as newly employed. The nurses went through various stages as they adapted to changes in their new professional role in a new context. The transition time varied and had no time limit, nor an average time as it depended on and differed between the newly employed nurses. The process involved three stages where a transition was described: the beginning, the middle, and the end of the introduction.

*At the beginning of the introduction*, the nurses described a transition that involved a sense of insecurity and confusion and placed high demands on their own ability when comparing themselves with their more experienced colleagues. Furthermore, putting on the uniform was described as putting on a costume, and then becoming an “*ambulance nurse”* by playing charades.

Then you can wear these clothes and hide behind them and pretend things. But from the beginning, it really was like putting on a costume.32:5.

*In the middle*, the newly employed nurses started feeling more secure in their role as their professional identity was forming. Their knowledge and skills improved day by day, and even though they still felt new, they started having fun and dared to “stay and play” rather than “load and go” the patients as fast as possible. However, the transition process was individual. While some nurses’ security had increased, some of the nurses still felt insecure and said that putting on the uniform still felt like acting. The newly employed nurses also described a temporary decline in their self-confidence and compared being new to the ambulance service to prior experience of being new in other contexts.

*At the end*, the newly employed nurses described being ready to work independently, and said they had a positive feeling and were looking forward to a sense of freedom when working without the supervisor. The newly employed nurses had developed a new identity which they described as a nurse working in the ambulance. At the end of the introduction, the nurses could reflect on their transition as going from uncertainty to security. There were however nurses who described not feeling ready to work independently at the end of the introduction. They were still uncertain and reflected that the sense of being new lasted longer in the ambulance service than it had when they were new at their previous workplaces.

*Yes, but it feels… it’s scary, it is. But it’s been fun. Trying to stand on your own two feet. But it took me a while to realise that it’s the case that… Yes, but it’s embarrassing because I don’t understand that I’m not in school now. But now I go on my own. It’s a bit unusual. But finally, I understood, when I was going to drive to the hospital and realised that I was sitting there alone. 21:1*.

### Critical points

This category includes various situations where the newly employed nurses encountered significant critical points and events that had a profound impact on the transition process into their new professional role. Critical points concerned situations when the newly employed nurses realised the importance of *becoming ready*, *becoming accepted, and becoming secure*. In terms of situations where the importance of *becoming ready* was recognised, this involved managing new situations as well as being exposed to situations the nurses were not ready for. Ending up in a new situation that felt challenging, and then realising that they were able to handle things using either prior knowledge or newly acquired knowledge had a positive effect on the nurse’s transition. On the other hand, being exposed to a situation that the nurses did not feel ready for created uncertainty and confusion. The outcome of such situations was described as affecting them by increasing uncertainty.

*”So then I felt “Okay, give yourself a pat on the back”. It was a difficult task, it was difficult. But it went well, we got to the hospital. So for my professional development, it was great. 11:9”*.

The sense of *being accepted* occurred when the supervisor gave honest feedback and highlighted the nurse’s strengths or just gave support, and it created a positive feeling for the nurses and the transition. Having a supportive supervisor who took responsibility for the nurse’s introduction and shared their knowledge and skills also had a positive impact on the transition. Furthermore, the sense of *becoming secure* was described as having personal chemistry with their colleague or the group; this made the nurses feel secure in their roles and their new tasks. Having a personal chemistry with colleagues created a sense of acceptance, a calm feeling, and a sense of security that together they could cope with the care of the patient and the assignments.

*”For my part, I think I probably got into this slightly calmer feeling more quickly because my colleague and I know each other and we have worked before and it feels kind of good, I feel confident that we can resolve situations. 29:4”*.

### Dealing with the ambulance culture

The transition into a new professional role in a new context is a complex process, encompassing various stages, challenges, and critical points that newly employed nurses must navigate. Additional findings from this study showed that newly employed nurses also had to contend with the influence of the ambulance culture, which appeared to play a significant role during their transition. Finding one’s place in the social culture at the ambulance station, as well as finding one’s place in the culture existing among colleagues was described as an important part of the transition. Becoming a part of the workgroup was described as a need to navigate and position oneself with colleagues, within the group at the station, and in the organisation. The sense of becoming a part of the workgroup was shown by the perceived need to accept and to adapt to the different attitudes, personalities, and how to perform the clinical work. The culture in the ambulance service was described as a mixture of friendly and harsh, and/or helpful and macho. In this dual culture, the newly employed nurses said that they needed to navigate and position themselves to clarify their own professionalism. To position themselves meant sometimes having to stand up to senior colleagues with deep-rooted working methods and values that the new nurses did not share. According to the nurses, the culture was described by the senior colleagues as an open-minded and relaxed atmosphere that facilitated giving feedback. However, the newly employed nurses perceived this relaxed atmosphere as an opportunity for the colleagues to be able to insult each other and express themselves meanly towards others; it was not only used from a constructive perspective. The newly employed nurses said they had a hard time becoming a part of the workgroup, which affected the time of the transition as they needed to adapt and position themselves in the culture.

*” A fairly clear factor in being new is that there are a lot of different people in the organisation. They talk about each other, I get to know people, I create my own ideas about people. And somewhere in there, the formal competence hierarchy is nothing strange, I’m quite confident there. But as for the people in the company, I’m still learning who I like, who I get along with, whose opinions I don’t like… and so… well, it´s people, so they talk about each other. I’m not saying that they talk a lot of crap about each other, that’s not what it’s about, but in the social context, you hang out and talk about each other and about the events you’ve been a part of. And there is a clear oral tradition where experiences are spread.19:7”*.

The nurses described ambulance professionals as independent and strong individuals used to making their own rapid and tough decisions, which according to the newly employed nurses built strong professional autonomy. The newly employed nurses stressed that since ambulance professionals hold the responsibility for decisions, they thereby possess a certain power over the patients and sometimes abuse that power. The newly employed nurses also experienced structural behaviour by some colleagues which was exemplified as wanting to leave the patient at home instead of following guidelines. In several cases, the assessment of patients was made before the ambulance arrived on the scene and as newly employed nurses it was described as difficult to speak up when not agreeing with a colleague’s assessment or if the behaviours did not accord with guidelines and one’s own values.

My feeling that this… it’s a structural behaviour. In whether you like to drive people in or not. I think that those who leave patients at home more often are confident in their cause, or at least I hope so. I have heard examples of colleagues not performing the procedure because of the risk of finding something…. We do not take an EKG because then we risk finding something abnormal. Then we can’t leave the patient at home. It’s typical bad jargon that I’ve identified. 19:9.

According to the newly employed nurses, different behaviours among colleagues had an impact on their transition and their development into the new profession. The newly employed nurses identified a risk that senior colleagues’ negative attitudes could spread and affect themselves and/or others in the organisation. In addition, the negative attitudes among colleagues could also create a discordant and sometimes incorrect image of the organisation. They reflected that it needs courage and a certain personality to deal with a colleague’s attitude without letting it affect oneself.

I absolutely believe that you are influenced by the person you ride with. Because we’re herd animals. We want to hang out, we want to get along. We don’t like to have conflicts. If you are new to an organisation, you are a little lower in the hierarchy, somehow. And it is clear, then there is a risk that you may acquiesce to behaviours that you may not think are completely okay until you are brave enough to stand on your own two feet. It depends a little on who you are as an individual.19:15.

## Discussion

To the best of our knowledge, this study is the first to explore the transition of newly employed experienced nurses as they develop a new professional role, using Meleis’ [15] transition theory. Although the duration of the transition varied among participants and for the nurses that had a transition, all phases of Meleis’ theory were identified for those who underwent a transition, implying that the theory can be used to explain new employees’ transition in the ambulance service. The nurses went through various situations, and the duration and experience of the transition varied among the nurses as they gradually adopted their new professional role. The time span is consistent with Meleis’ theory [15] that a transition is individual and is a process that takes place over time, and for some, it is not straightforward.

Being new can be both scary and challenging [4]. The fear of failure or not meeting the demands and expectations of the employer and colleagues can be particularly challenging [7, 34]. Meleis [15] states that to be in transition, the individual needs to be aware of the changes and engage in the process. Some individuals struggle to balance the expected development with the actual development during the transition. Helping newly employed nurses to be conscious of their own awareness may contribute to better adaptation and development of different coping strategies in their new profession. Mentorship or collegial reflections could be a way to help the newly employed nurses to become conscious of their transition. It has been shown that senior supervisors and mentors have a positive influence on nurses’ transition provided they have a good attitude [35], and that feedback from the workgroup can promote professional development for the nurses and contribute to a change in clinical performance [36], but further research is needed to address this.

In this study, the newly employed nurses described being new as extremely challenging and they described different learning conditions that were supportive during their transition. Learning conditions used were to display humbleness about being new and having self-confidence in their own learning. The newly employed nurses said that at the beginning of the introductory training, they felt that being an “ambulance nurse” was like playing charades. Even though the environment was welcoming, they felt insecure and experienced that high demands were put on them. These conflicting emotions are in line with previous research [11–13]. One commonly occurring aspect of being new is having conflicting emotions, such as being insecure and still having to act confidently [37]. These findings may suggest that the management of organisations need to be more aware of the process of transition, and perhaps a systematic evaluation of the professional development of newly employed nurses during the first year is needed to facilitate their transition into a new profession.

In line with Meleis transition theory [15], our findings showed that the transition for newly employed nurses is a complex process involving various stages and that the nurses needed to identify their new professional role and establish stability in their new working environment and contextual culture. Being an experienced nurse new in a new context can be a period of uncertainty and frustration, but helping the newly employed nurses towards a healthy transition may result in increased self-confidence after the first year of employment, reduced turnover, and improved patient safety. However, further research is needed to confirm if this assumption holds true. Regardless, critical points during the transition process were identified as situations when newly employed nurses recognised the significance of readiness, acceptance, and security while entering their new professional role. Drawing upon the theory of communities of practice [38], as well as the desire of new employees to feel a sense of belonging to a group [4], and previous research on peer learning [39], an assumption is that implementing peer learning during the induction training could support new employees in developing a sense of belonging and security within the group. By using peer learning in the ambulance service during introductory training it could be possible to create groups that have a balanced composition of new employees and supervisors. Peer learning may also positively influence the transition for the newly employed as it is known that an individual’s transition can be affected by others [19, 20, 40]. However, further research is needed to investigate if peer learning could support the newly employed and their supervisors.

Out of our findings, adapting to the ambulance culture appears to be a sometimes challenging aspect of the transition for newly employed nurses into the new professional role. These findings correspond to previous reports showing that ambulance personnel have their own expectations, both socially and within the profession itself [41, 42]. The newly employed also described how senior colleagues insulted each other and expressed themselves meanly towards others. Bullying and a harsh work environment are described to be relatively common in healthcare, which is a systemic problem and will not disappear overnight. Bullying behaviour transcends age, gender, and experience level. However, research indicates that senior nurses, who have spent more time in the same workplace, may take advantage of the unfamiliarity and lack of experience of their younger colleagues in the new work environment to portray themselves as more proficient [43]. Whether the experiences of the newly employed constitute bullying is unclear, but for a healthy transition to be possible, there must be a zero-tolerance policy towards bullying [43]. Furthermore, our findings imply the importance of supporting newly employed nurses by reinforcing their values, rather than adopting a negative social culture with the ambulance service. Even though the newly employed nurses in this study described the significance the ambulance culture had for their transition, this study does not provide a clear understanding of how the social culture within the ambulance service influences new employees’ professional role development. Further research is necessary to explore the culture and interactions within the ambulance service.

It was also found in this study that not all nurses described a transition during their first six months in accordance with Meleis’ transition theory. The reasons for this are unclear and need further research. But based on Hallaran’s research [44], both the organisation and colleagues play a crucial role in the transition process for nurses in a new workplace. A lack of effective transition can impact a nurse’s decision to resign. Additionally, inadequate preparation during the transition process is linked to a higher turnover rate [45]. Regardless, it is essential for healthcare organisations and colleagues to enhance their understanding of the effects of transition and how a well-managed transition can contribute to higher retention rates in the workplace.

### Strengths and limitations of the work

The use of a qualitative approach facilitated a comprehensive description of the transition process experienced by newly employed experienced nurses in the ambulance service. Another strength is the data collection; conducting interviews over six months allowed us to explore the nurses’ transition into a new working environment. The data collection was carried out by two of the authors, and before and after the interviews, there were continuous discussions to maintain a cohesive approach among the interviewers. However, the study’s data collection also poses limitations, as it was restricted to one of the ambulance organisations contracted by the region and the introduction programme may have differed between the ambulance organisations despite the provided framework by the region. Newly employed experienced nurses in diverse locations may have varied experiences, potentially limiting the findings’ transferability. Despite this, the goal was to explore the transition of newly employed experienced nurses, a phenomenon that is probably transferable to other ambulance service contexts. The worldwide similarity of ambulance services in providing care for accident victims and those with acute illnesses, irrespective of predetermined care environments [46], supports the assumption that our findings could be applicable to other ambulance service organisations. In addition, the findings could also have relevance and applicability to other ambulatory care settings where nurses operate independently and without predefined care environments.

Regarding the newly employed nurses’ demographics, it is worth noting that 83% of the newly employed nurses were women. This homogeneity could have restricted the diversity of the data. However, given the current trend of increasing female employment in the ambulance service [47], it is reasonable to assume that the participants reflect the presence of employees. All authors, three females, and one male, had substantial experience of working as SNs in the ambulance service, which may have introduced biases during the study design, data collection, and analysis. However, of the authors, three were trained in qualitative study design and the fourth author was a PhD student. To reduce the risk of bias, the analysis process involved critical discussions with researchers outside the author team, providing an outside perspective. Despite the potential for bias, the authors’ preconceptions may also serve as a strength in interpreting and understanding the study findings [48].

The employed theory and the authors’ preconceptions concerning the study context and their personal experiences have partially influenced the interpretation of the collected data. During the interviews, some informants said that the interviews allowed them to reflect on their transition with a senior colleague who was not their supervisor. This may have influenced the data collection in two ways; either because the participants increased their learning from the introductory training and/or because they gained a deeper understanding of their own transition. Either way, both interviewers agree that these reflections increased the depth of data.

### Implications for policy and practice

The findings of the study can be utilised for the development of introductory training for newly employed experienced nurses. By using the knowledge generated from this study, managers, colleagues, and supervisors can provide support to newly employed nurses during their transition to a new workplace and professional development. By acknowledging and incorporating the experiences of newly employed nurses, the organisational culture can be brought to light and further enhanced.

## Conclusion

This study shows that the transition process experienced by newly employed experienced nurses in the ambulance service can be understood/described using Meleis’ transition theory. The findings highlight the importance of understanding the impact that context may have on a new employee’s transition process. Furthermore, this study emphasises the need for tailored introductory training for newly employed nurses with previous experience as RNs or SNs when entering the ambulance culture. Understanding how newly employed nurses transition into their new role and how the contextual culture affects the transition can help ambulance organisations improve introduction programmes, support newly employed nurses, decrease the turnover rate, and increase patient safety. Further research is needed to explore how the organisational culture affects professional development among RNs and SNs entering a new context.

## Relevance to clinical practice

The findings from this study provide insights into the necessity of developing introductory training programmes for new employees that also take into account their prior experience as RNs and SNs in other contexts. It is therefore imperative for healthcare organisations to establish ways to facilitate the transition for newly employed nurses into their new professional roles in a new context. This may make the expectations of new employees more realistic, by creating supportive environments for the nurses to navigate through during their transition process and by offering educational resources for the supervisors.

## Data Availability

The datasets used and/or analysed during the current study are available from the corresponding author on reasonable request.
